# Human Papillomavirus Genotyping and E6/E7 mRNA Expression in Greek Women with Intraepithelial Neoplasia and Squamous Cell Carcinoma of the Vagina and Vulva

**DOI:** 10.1155/2012/893275

**Published:** 2011-12-05

**Authors:** Elpida Tsimplaki, Elena Argyri, Lina Michala, Maria Kouvousi, Aikaterini Apostolaki, George Magiakos, Issidora Papassideri, Efstathia Panotopoulou

**Affiliations:** ^1^Department of Virology, “G. Papanicolaou” Research Center of Oncology and Experimental Surgery, Regional Anticancer Oncology Hospital of Athens “St. Savvas”, 171 Alexandras Avenue, 11522 Athens, Greece; ^2^1st Department of Obstetrics and Gynecology, Alexandra Hospital, University of Athens, Vas Sofias 80, 11528 Athens, Greece; ^3^Department of Pathology, Regional Anticancer Oncology Hospital of Athens “St. Savvas”, 171 Alexandras Avenue, 11522 Athens, Greece; ^4^1st Department of Gynecology, Regional Anticancer Oncology Hospital of Athens “St. Savvas”, 171 Alexandras Avenue, 11522 Athens, Greece; ^5^Department of Cell Biology and Biophysics, Faculty of Biology, University of Athens, Panepistimiopolis, 15784 Athens, Greece

## Abstract

A large proportion of vaginal and vulvar squamous cell carcinomas (SCCs) and intraepithelial neoplasias (VAIN and VIN) are associated with HPV infection, mainly type 16. The purpose of this study was to identify HPV genotypes, as well as E6/E7 mRNA expression of high-risk HPVs (16, 18, 31, 33, and 45) in 56 histology samples of VAIN, VIN, vaginal, and vulvar SCCs. HPV was identified in 56% of VAIN and 50% of vaginal SCCs, 71.4% of VIN and 50% of vulvar SCCs. E6/E7 mRNA expression was found in one-third of VAIN and in all vaginal SCCs, 42.9% of VIN and 83.3% of vulvar SCCs. Our data indicated that HPV 16 was the commonest genotype identified in VAIN and VIN and the only genotype found in SCCs of the vagina and vulva. These findings may suggest, in accordance with other studies, that mRNA assay might be useful in triaging lesions with increased risk of progression to cancer.

## 1. Introduction

Human papillomavirus (HPV) infection of the female genital tract, is particularly frequent worldwide and its majority is transient, while at the same time, the persistent infections caused by the oncogenic types of HPV are responsible for cancer development. This oncogenic action of HPV is a result of the transformation ability of the high-risk (hr) HPV types' oncoproteins E6 and E7. The oncogenic properties of high-risk HPV reside in the E6 and E7 genes, which if inappropriately expressed in dividing cells deregulate cell division and differentiation [1].

The causal relation of HPV infection with development of cervical cancer has been firmly established. The same does not necessarily apply to vulvar and vaginal squamous cell carcinomas (SCCs), where HPV is responsible for only a smaller percentage, namely, 60–70% of vaginal SCCs [2, 3] and 38–75% of vulvar SCCs [2, 4]. Interestingly, HPV infection is strongly associated with intraepithelial neoplasia of the vagina (VAIN) and vulva (VIN) (93.6% and 84%, resp.) [5]. HPV 16 is by far the commonest HPV type identified in vaginal and vulvar SCCs and precancerous lesions [6]. The above data may vary from population to population, and to our knowledge, no such analysis has been conducted in Greece.

The purpose of the current study was the HPV genotyping, as well as the expression of E6/E7 mRNA from the hrHPV types (16, 18, 31, 33, and 45) in Greek women with VAIN, VIN, and SCCs of the vagina and vulva.

## 2. Materials and Methods

### 2.1. Study Population and Collection of Specimens

The sample of this study consisted of 56 paraffin-embedded tissue sections of VAIN, VIN, or vaginal and vulvar SCCs obtained from the Pathology Laboratory of the Regional Anticancer Oncology Hospital of Athens “St. Savvas”. The tissues were deparaffinized, and total nucleic acid was extracted using automated extraction (NucliSENS easyMAG, bioMérieux Hellas S.A). Then, genotyping of 24 HPV types was performed with microarray-based assay (PapilloCheck HPV-Screening, Greiner Bio-One GmbH, Germany, cat no. 465 060) as well as the expression of HPV 16, 18, 31, 33, and 45 E6/E7 mRNA, using the commercial real-time NucliSENS EasyQ assay (NucliSENS EasyQ HPV 1.1, bioMérieux Hellas S.A, cat no. 290003).

Although VIN terminology was changed by the International Society for the Study on Vulvovaginal Disease in 2004, we maintained the original terminology which was used during the histological diagnosis [6].

Ethical approval was granted by the ethics committee of the Regional Anticancer Oncology Hospital of Athens “St. Savvas”, and all participants provided written informed consent.

### 2.2. Deparaffinization

Fifteen micrometer sections of paraffin-embedded tissues were deparaffinized by incubation with 100% xylene (Applichem GmbH, Darmstadt, Germany, cat no. 10-20/21-38) at 50°C and washings twice with 100% ethanol (Applichem GmbH, Darmstadt, Germany, cat no. 64-17-5). Then, the pellets were dried at room temperature for 45 minutes. Finally, deparaffinized samples were digested with 100 *μ*L digestion buffer [1 mL TE buffer 1X (Invitrogen corp., Calif, USA, cat no. 12090-015) and 5 *μ*L 50% Tween20 solution (Invitrogen corp., Calif, USA, cat no. 00–3005)] and 5 *μ*L proteinase K solution (Invitrogen corp., Calif, USA, cat no. 25530-049) at 65°C overnight. Proteinase K was deactivated in heat block at 80°C for 15 minutes.

### 2.3. Extraction of Nucleic Acids

Tissue samples were transferred in lysis buffer (NucliSENS lysis buffer, bioMérieux Hellas S.A, cat no. 200292) for 30 minutes, then total nucleic acid was extracted by the off-board protocol with the NucliSENS easyMAG platform (bioMérieux Hellas S.A), according to the manufacturer's instructions. The nucleic acids were eluted in 55 *μ*L of elution buffer. DNA quality test was carried out using Human Globin, Beta, Primer set kit (Maxim Biotech, Inc., South San Francisco, CA) according to manufacturer's instructions. To assess RNA integrity, 5 *μ*g of RNA per sample was separated on 1% formaldehyde-agarose gel.

### 2.4. HPV Genotyping (PapilloCheck HPV DNA Microarray)

The PapilloCheck HPV-Screening was used. This technology is based on a DNA chip for the type-specific identification of 24 types of HPV (18 high-risk and 6 low-risk types). E1-based PCR was performed according to the manufacturer's guidelines. For each sample, we mixed 19,8 *μ*L PapilloCheck MasterMix, 0,2 *μ*L HotStarTaq plus DNA polymerase (5 U/*μ*L, Qiagen, cat no. 203605), and 5 *μ*L DNA from the tissue sample. Hybridization is followed by mixing 30 *μ*L of the PapilloCheck hybridization buffer in a fresh reaction tube with 5 *μ*L of the PCR product at room temperature and transferring 25 *μ*L of the hybridization mix into each compartment of the chip. We incubated the chip for 15 minutes at room temperature in a humid atmosphere. The chip was washed in 3 washing solutions, centrifuged for 3 minutes at 5000 rpm, and scanned on the CheckScannerTM.

### 2.5. HPV E6/E7 mRNA Expression (NucliSENS EasyQ HPV Assay)

A commercial real-time NucliSENS *EasyQ* assay (NucliSENS EasyQ HPV 1.1, bioMérieux Hellas S.A, cat no. 290003) was performed for the qualitative detection of HPV E6/E7 mRNA of five hrHPV types (16, 18, 31, 33, and 45) according to the manufacturer's instructions. Firstly, three premixes were made by adding reagent sphere diluent (Tris-HCl, 45% DMSO) into reagent spheres (nucleotides, dithiotreitol, and MgCl2). In each premix, we added U1A/HPV 16, HPV 33/45, or HPV 18/31 primer and molecular beacon mixes, KCl stock solution, and NASBA water. Secondly, 10 *μ*L of this premix was distributed to each well in a reaction plate, and the addition of 5 *μ*L RNA followed. The plates were incubated for 4 minutes at 65°C to destabilize secondary structures of RNA, followed by cooling down to 41°C. The reaction was started by addition of enzymes (AMV-RT, RNase H, T7 RNA polymerase, and bovine serum albumin) and measured in real time using the Lambda FL 600 fluorescence reader (Bio-Tek, Winooski, VT) at 41°C for 2 hours and 30 minutes.

### 2.6. Statistical Analysis

Our data were analyzed using SAS v9.0. Absolute and relative frequencies were used to present the HPV prevalence according to age and histology. Chi-squared tests were performed to assess the statistical significance of any differences in prevalence. *t*-test was used, along with relevant descriptive statistics (mean value, standard deviation, and 95% confidence interval for the mean value) to compare the average age among patients depending on the prevalence of HPV and histology. In all the statistical tests, 5% level of significance was used. Concordance between DNA and mRNA tests was evaluated using the Cohen's kappa statistic.

## 3. Results

### 3.1. Vaginal Intraepithelial Neoplasia and Vaginal Squamous Cell Carcinoma

This group consisted of 18 patients with VAIN (mean age 46.2 years) and 4 patients with SCCs (mean age of 61.3 years). 8 VAIN cases were classified as VAIN I and 10 as VAIN II/III. HPV infection was detected in 56% of VAIN (10/18) and in 50% of vaginal SCCs (2/4). Detectable HPV DNA from at least one of the 24 genotypes was found in 75% (6/8) of cases of VAIN I and 40% (4/10) of VAIN II/III. In VAIN cases, the presence of HPV infection was strongly associated with younger patient age (38.7 versus 55.6 years, *P* = 0.006).

hrHPV types were found in 70% (7/10) and lrHPV types in 30% (3/10) of VAIN cases. Only hrHPVs were detected in vaginal SCCs (2/2). Multiple HPV types were present only in one case (one VAIN II/III sample contained HPV 33 and 53).

The commonest HPV genotype was HPV 42 for VAIN I samples (3/6 cases, 50%), followed by HPV 16 (2/6 cases, 33.3%) and HPV 39 (1/6 cases, 16.7%), while HPV16 and HPV33 were the most common types for VAIN II/III (2/4 cases, 50% each). HPV 16 was the only type associated with HPV-infected vaginal SCCs (2/2 cases, 100%).

**Figure 1:**
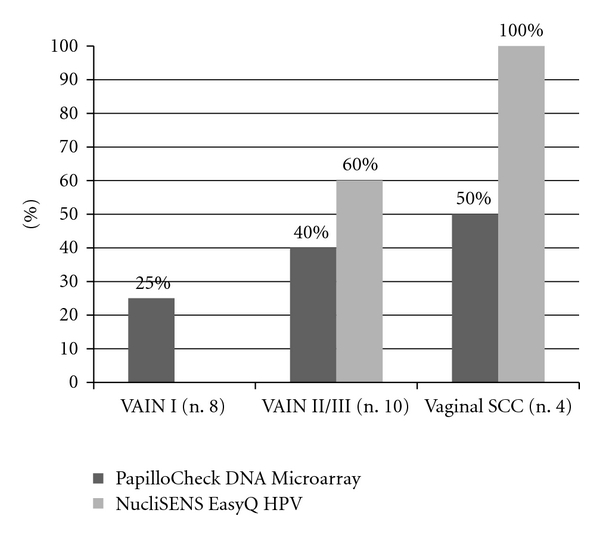
Prevalence of the hr HPV genotypes (16, 18, 31, 33, and 45) detected by both PapilloCheck DNA Microarray and NucliSENS EasyQ HPV assay according to histological status of samples of vagina.

Regarding the hrHPV types 16, 18, 31, 33, and 45, 33.3% (6/18) of the VAIN and 50% of vaginal SCCs were HPV DNA positive for at least one of the above types. The detection rates of the five hrHPV types in samples from women with different grades of VAIN and SCCs are provided in Figure 1.

All vaginal SCCs (4/4) and 60% (6/10) of VAIN II/III were positive for HPV E6/E7 mRNA expression. This was statistically significantly higher than VAIN I samples, where no HPV E6/E7 mRNA expression was detected (*P* = 0.01 and *P* = 0.002), respectively (Figure 1).

The expression pattern for hrHPV types 16, 18, 31, 33, and 45, according to VAIN grade and SCCs, is summarized in Table 1.

The concordance between the HPV DNA test (PapilloCheck DNA Microarray) and HPV E6/E7 mRNA test (NucliSENS EasyQ HPV assay) results was poor for patients with VAIN I (75%; kappa = 0.00) and vaginal SCCs (50%; kappa = 0.00), whereas it was good for patients with VAIN II/III (80%; kappa = 0.62) (Table 2).

### 3.2. Vulvar Intraepithelial Neoplasia and Vulvar Squamous Cell Carcinoma

We included 28 patients with VIN, half of which had a low-grade lesion (mean age 35 years) and 6 patients with vulvar SCCs (mean age 62.5 years). VIN cases were not associated with lichen sclerosus, differentiated vulvar intraepithelial neoplasia, or squamous cell hyperplasia. PapilloCheck DNA Microarray detected HPV infection in 71.4% of VIN samples (20/28) and in 50% of vulvar SCCs (3/6). HPV DNA from at least one of the 24 genotypes was detected in 64.3% (9/14) of women with VIN I, and 78.6% of those with VIN II/III (11/14). In VIN cases, the presence of HPV infection was associated with younger patient age (31.7 versus 43.1 years, *P* = 0.001).

PapilloCheck assay detected hrHPV infection in all VIN cases and vulvar SCCs. Multiple infection was detected in 5 cases (3 VIN I cases contained HPV 6/16, HPV 6/16/59 and 11/59, resp., and 2 VIN II/III cases contained HPV 16/53 and HPV 16/51/66, resp.).

HPV16 and HPV59 were the most commonly observed for infected VIN I samples (4/9 cases each and one case with multiple infection HPV 6/16/59, 55.6% each), while HPV16 was the commonest genotype for VIN II/III (9/11 cases, 81.8%) followed by types 18, 51, 52, 53, and 66 (1/11 cases, 9.1% each). In HPV-positive vulvar SCCs, only HPV 16 was present (3/3 cases, 100%).

**Figure 2:**
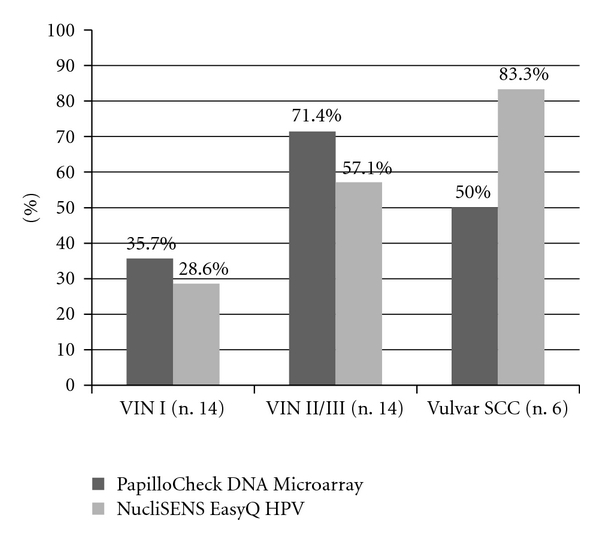
Prevalence of hr HPV genotypes (16, 18, 31, 33 and 45) detected by both PapilloCheck DNA Microarray and NucliSENS EasyQ HPV assay according to histological status of samples of vulva.

Regarding the hrHPV types 16, 18, 31, 33, and 45, 53.6% (15/18) of VIN and 50% of vulvar SCCs were HPV DNA positive for at least one of the above HPV types by PapilloCheck DNA Microarray. The detection rates of the five hrHPV types in samples from women with different grades of VIN and SCCs are provided in Figure 2.

42.9% of VIN samples (12/28) and 83.3% of vulvar SCCs (5/6) were positive for HPV E6/E7 mRNA expression. There was a higher prevalence of E6 and E7 mRNA expression in patients with higher-grade lesions as shown in Figure 2. The difference in detection of E6/E7 mRNA expression between VIN I and vulvar SCCs was statistically significant (*P* = 0.04).

HPV16 was the commonest type revealed by NucliSENS EasyQ HPV assay for VIN I, VIN II/III, and vulvar SCCs (4/4 cases: 100%, 6/8 cases: 75%, and 3/5 cases: 60%, resp.) (Table 1).

The concordance between the HPV DNA test (PapilloCheck DNA Microarray) and HPV E6/E7 mRNA test (NucliSENS EasyQ HPV assay) results was very good for samples classified as having VIN I (92.9%; kappa = 0.84) but was poor for patients with VIN II/III (57.1%; kappa = 0.09). In the case of samples classified as having vulvar SCCs, the concordance was fair (66.7%; kappa = 0.33) (Table 2).

## 4. Discussion

The goal of this study was to investigate HPV typing and HPV E6/E7 mRNA expression with intraepithelial neoplasia and squamous cell carcinomas of the vagina and vulva. This is the first study to report the association between HPV infection with oncogenic expression and vulvovaginal disease in Greek women. 

The role of HPV infection in vulvar intraepithelial neoplasia and squamous cell carcinoma has been confirmed through multiple studies worldwide. Although a similar link exists in vaginal precancerous and cancerous lesions, this has not been firmly established. This in part is due to the fact that VAIN and vaginal carcinomas are less common than their vulvar and cervical counterparts, due to the absence of a susceptible transformation zone and the protective effect of the keratinized vaginal mucosa [7]. Furthermore, it is possible that a proportion of VAIN lesions remain undiagnosed as they are asymptomatic and not easy to visualize during a routine gynecological examination [5]. Nevertheless, we expect an outbreak of VAIN and VIN cases, as well as SCCs of the vulva and vagina, especially in younger women[8, 9]. This is thought to be due to not only the rapid spread of HPV, but also the increased gynecological surveillance and improved diagnostic techniques, aimed at the identification of cervical lesions.

As mentioned above, several studies have previously described the HPV prevalence and genotype distribution in VIN and SCCs of the vulva [3, 4, 10–16]. The results of the current study are generally similar to worldwide VIN and vulvar SCCs data although some differences were observed. In our study, the overall HPV prevalence was 71.4% in VIN and 50% in vulvar carcinoma. A recent international meta-analysis of 14 studies on vaginal and 63 studies on vulvar lesions reported that the overall HPV prevalence in VIN was 84.0% and 40.4% in vulvar SCCs [5]. Similar results were reported by other studies which indicated HPV prevalence of 79.6% in VIN and 40.1% in vulvar SCCs [15, 16] (Table 4).

According to our results, hrHPV infection was the most frequently observed in VIN I cases. This is contrary to data obtained from an older study [3], where low-risk (lr) HPVs were the commonest types. HPV 16 and HPV 59 were the two most frequent genotypes in VIN I, accounting for 35.7%, whereas in the recent international meta-analysis HPV 6 was found to be the commonest genotype [5]. HPV 16 was by far the commonest type in VIN II/III and vulvar SCCs in our study, accounting for 64.3%, similar to other reports [4, 14] (Table 4).

35.7% of VIN I samples harbored hrHPV (16, 18, 31, 33, and 45) and this rose to 71.4% in VIN II/III samples. This is expected as women with hrHPV infection are more likely to progress to high-grade lesions. Interestingly, the percentage of hrHPV dropped to 50% in vulvar SSCs. This suggests that the virus was present only at very low copy numbers and/or that only a specific region of viral DNA was integrated into the host's genome [17].

In our study, HPV prevalence in VAIN samples was 56%. This was significantly lower than those reported by De Vuyst et al. (93,6%) and Smith et al. (95.6%). In vaginal SCCs, HPV prevalence was 50%. This was lower but more comparable to those found in the studies mentioned above (65.5% and 69.9%, resp.) [5, 15] (Table 3).

In a worldwide meta-analysis, the most frequent genotypes were HPV 16, 56, and 51 in patients with VAIN I lesions, HPV 16, 18, and 58 in those with VAIN II/III, and HPV 16, 18, and 31 in those with vaginal SCCs [5] (Table 3). In our study, we detected, in decreasing order, HPV 42, 16, and 39 in VAIN I cases, HPV 16, 33, and 53 in VAIN II/III, cases, and HPV 16 in SCCs. It is interesting to note that HPV 18 was not identified in any of our VAIN or SCC samples, as opposed to what is seen elsewhere, and this may be a geographical variation that needs to be investigated further. Another variation, possibly attributed to our different sample population, was that HPV 42, a lrHPV type, was the most frequently identified type in VAIN I, whereas in the studies by De Vuyst et al. and Smith et al., no lrHPVs were detected in low-grade lesions [5, 15].

Contrary to what was observed in vulvar samples, the proportion of patients with detectable hrHPV (16, 18, 31, 33, and 45) increased progressively as the grade of the vaginal lesion progressed.

Using the NucliSens EasyQ HPV assay, we investigated HPV oncoprotein expression in different grades of dysplasia and carcinoma. The results from E6/E7 mRNA test related well with the grade of lesion. The lowest rates of hrHPV types (16, 18, 31, 33, and 45) E6/E7 mRNA expression were for patients with low-grade vulvar lesions (VIN I), whereas the higher rates were seen in high-grade lesions (VIN II/III), which is in accordance with what is seen in cervical lesions [18–20]. It is possible that VIN I lesions that have detectable E6/E7 mRNA expression are the ones with a potential to progress to higher-grade lesions and malignancy, and therefore, E6/E7 mRNA expression could be used as a screening marker for better surveillance in this subcategory of women. Interestingly, none of the five high-risk types E6/E7 mRNA expression was detected in low-grade vaginal lesions (VAIN I). The above findings probably suggest that VAIN I may not be strictly a precancerous disease, while at the same time, they reflect the transient nature of most HPV infections. On the contrary, VIN II/III, VAIN II/III, and vaginal and vulvar SCCs showed a high prevalence of E6/E7 mRNA expression. For VIN II/III in particular, the rates of E6/E7 mRNA expression that we found were significantly higher than what has been published previously (57.1 versus 38.1%) [21].

In our series, there was a significantly higher detection of HPV 16 by NucliSens EasyQ HPV assay when compared to other hrHPV types for both vaginal and vulvar cases. This indicates that HPV 16 may be related to a different nature of persistent infection and oncoprotein expression in the vagina and vulva in comparison to HPV types 18, 31, 33, and 45.

On the basis of DNA and mRNA assays, DNA from HPV was detected more frequently in vulvar low-grade lesions than E6/E7 mRNA expression. This data possibly reflects an episomal state or low number of copies of the virus. However, E6/E7 mRNA expression in a number of VIN I indicates that hrHPV may be oncogenically active even before it produces detectable changes in the cell [17].

For vaginal high-grade lesions and carcinomas, as well as vulvar carcinomas, a higher rate of E6/E7 mRNA expression was observed, compared to HPV DNA. This suggests that the presence of E6/E7 oncoproteins is a specific marker for high-grade lesions. Interestingly, 2 cases of VAIN II/III, 1 case of VIN II/III, 2 cases of vaginal SCCs, and 2 cases of vulvar SCCs were positive only for mRNA expression. This may be explained by the fact that total viral DNA has been integrated to the host genome, and therefore, it cannot be detected by the DNA test. It is important to notice that in vulvar high-grade lesions a higher detection rate for HPV DNA was observed compared to E6/E7 mRNA expression. It is possible that these results were due to a very low level of viral transcriptional activity.

The sample in our study was relatively small, primarily due to the low incidence of vulvar and vaginal intraepithelial neoplasias and carcinomas. Thus, we can only provide a rough estimate of the relative importance of each HPV type with regards to vaginal and vulvar cancer and precancer in our population.

Another limitation of our study was that histology types of vulvar SCCs were not available, so a correlation between histology types and HPV infection was not possible. Nevertheless, it has been suggested that differentiated keratinizing SCCS, which occurs more frequently in elderly women, is not associated with HPV infection, whereas nonkeratinizing SCCS, which primarily affects younger women, is caused by hrHPV infection [22]. This was indirectly confirmed in our study, as HPV infection was more likely to be found in younger women.

## 5. Conclusion

This study described the detection rates and attribution of genital HPV types, as well as the E6/E7 mRNA expression of intraepithelial neoplasias and squamous cell carcinomas of the vagina and vulva in Greek women. In summary, our results showed that a very crucial percentage of HPV was associated with VIN, VAIN, and vaginal/vulvar SCCs, and HPV 16 accounted for most HPV-positive cases. The fact that some cases of vulvar low grade lesions were positive for E6/E7 mRNA expression is also of interest, as it may identify these lesions as more clearly precancerous. A striking increase especially in the incidence of VIN in young women has been reported in the last decades in some high-resources countries [8, 23, 24].

Further research is required to better assess the role of mRNA testing as a molecular marker for vaginal and vulvar carcinogenesis.

## Figures and Tables

**Table 1:** Distribution of different HPV types detected by NucliSENS EasyQ HPV assay.

Histology result	HPV types				
	HPV16	HPV18	HPV31	HPV33	HPV45
VAIN I					
VAIN II/III	4			2	
Vaginal SCCs	4				
Total (*n*.10)	**8**			**2**	

VIN I	4				
VIN II/III	6				2
Vulvar SCCs	3			2	
Total (*n*.17)	**13**			**2**	**2**

Distribution of the five high-risk HPV types determined by NucliSENS EasyQ HPV assay in the vaginal and vulvar tissue samples.

**Table 2:** Concordance between HPV DNA test (PapilloCheck DNA Microarray) and HPV E6/E7 mRNA test (NucliSENS EasyQ HPV assay) by histological status of samples.

	No. of specimens	Number of specimens positive with		Concordance*	%	Kappa value	*P*
		HPV DNA test	E6/E7 mRNA test				
VAIN I	8	2	0	6/8	75.0	0.00	1.00
VAIN II/III	10	4	6	8/10	80.0	0.62	0.08
Vaginal SCCs	4	2	4	2/4	50.0	0.00	1.00
VIN I	14	5	4	13/14	92.9	0.84	0.005
VIN II/III	14	10	8	8/14	57.1	0.09	1.00
Vulvar SCCs	6	3	5	4/6	66.7	0.33	1.00

*The data represent the number of samples for which the results from PapilloCheck DNA Microarray and NucliSENS EasyQ HPV assay were concordant/total number of samples tested.

**Table 3:** Prevalence of HPV in intraepithelial neoplasia and carcinoma of the vagina, by study.

Histologic type	First author	No. of cases	HPV test	HPV prevalence for any and specific genotypes
VAIN I				
	Smith (review)	66	PCR/hybrid capture assays	Any HPV, 98.5% Most common types; 16 (17.9%), 18 (17.9%)
	De Vuyst (meta-analysis)	107	PGMY reverse line blot/SPF 10 line probe assay, blot hybridization	Any HPV, 100% Most common types; 16 (23.4%), 56 (11.0%), 51 (8.8%)

VAIN II/III				
	Smith	166	PCR/hybrid capture assays	Any HPV, 92.6% Most common type; 16 (65.8%)
	De Vuyst	191	PGMY reverse line blot/SPF 10 line probe assay, blot/southern hybridization, restriction fragment analysis, sequencing	Any HPV, 90.1% Most common types; 16 (57.6%), 18 (6.9%), 58 (5.9%)

Vaginal SCC				
	Smith	83	PCR/hybrid capture assays	Any HPV, 65.5% Most common type; 16 (55.4%)
	De Vuyst	136	Reverse line blot assay, INNO-LiPA HPV genotyping, southern hybridization, restriction fragment analysis, sequencing	Any HPV, 69.9% Most common types; 16 (53.7%), 18 (7.6%), 31 (5.6%)

**Table 4:** Prevalence of HPV in intraepithelial neoplasia and carcinoma of the vulva, by study.

Histologic type	First author	No. of cases	HPV test	HPV prevalence for any and specific genotypes
VIN I				
	Smith (review)	71	PCR/hybrid capture assays	Any HPV, 77.5% Most common types; 6 (23.8%), 16 (14.3%), 56 (1.7%)
	De Vuyst (meta-analysis)	90	PGMY reverse line blot/SPF 10 line probe assay	Any HPV, 67.8% Most common types; 6 (22.4%), 16 (9.8%), 11 (9.0%)
	Garland (original article)	31	Reverse line blot assay	Any HPV, 80.6% Most common types; 6 or 11 (64.5%)

VIN II/III				
	Smith	1340	PCR/hybrid capture assays	Any HPV, 80.4% Most common types; 16 (71.2%), 33 (7.7%)
	De Vuyst	1061	PGMY reverse line blot/SPF 10 line probe assay, sequencing, southern hybridization, restriction fragment-length polymorphism analysis	Any HPV, 85.3% Most common types; 16 (71.9%), 33 (8.0%),18 (5.0%)
	Garland	31	Reverse line blot assay	Any HPV, 87.1% Most common types; 16 (64.5%), 6, or HPV11 (29%)

Vulvar SCC				
	Smith	1379	PCR/hybrid capture assays	Any HPV, 40.1% Most common types; 16 (29.3%), 18 (5.6%)
	De Vuyst	1873	Reverse line blot hybridization, dot blot hybridization, Roche HPV linear array	Any HPV, 40.4% Most common HPV types; 16 (32.2%), 33 (4.5%), 18 (4.4%)
